# *Drosophila* integrin adhesion complexes are essential for hemocyte migration in vivo

**DOI:** 10.1242/bio.20134564

**Published:** 2013-06-06

**Authors:** Carolina G. A. Moreira, Antonio Jacinto, Soren Prag

**Affiliations:** 1 Instituto de Medicina Molecular, Faculdade de Medicina da Universidade de Lisboa, 1649-028 Lisboa, Portugal; 2 CEDOC, Faculdade de Ciências Médicas, Universidade Nova de Lisboa, 1169-056 Lisboa, Portugal; ‡ Present address: Carl Zeiss Microscopy GmbH, Königsallee 9-21, 37081 Göttingen, Germany

**Keywords:** *Drosophila*, Hemocyte, Integrin, Migration

## Abstract

Cell migration is an important biological process which has been intensively studied in the past decades. Numerous techniques, mainly involving two-dimensional cell culture systems, have contributed to dissecting the essential mechanisms underlying this process. However, the development of three-dimensional cell culture and in vivo systems has shown some differences with what was previously believed to be well-established cell migration mechanisms, suggesting that two-dimensional cell motility would be a poor predictor of in vivo behaviour. *Drosophila* is a widely recognized model organism to study developmental and homeostatic processes and has been widely used to investigate cell migration. Here, we focus on the migration of small groups of pupal hemocytes that accumulate during larval stages in dorsal patches. We show that integrins, and other known nascent adhesion-related proteins such as Rhea and Fermitin 1, are crucial for this process and that their depletion does not affect polarization in response to environmental cues. We also present evidence for the importance of adhesion maturation-related proteins in hemocyte migration, namely Zyxin. Zyxin depletion in hemocytes leads to a significant increase of cell speed without affecting their response to a chemotactic cue. This is the first report of a systematic analysis using *Drosophila melanogaster* hemocytes to study adhesion-related proteins and their function in cell migration in vivo. Our data point to mechanisms of cell migration similar to those described in three-dimensional in vitro systems and other in vivo model organisms.

## Introduction

Cell migration is a key mechanism that occurs during developmental and homeostatic processes such as tissue repair, immune surveillance and morphogenesis. The basic mechanisms underlying cell migration have been intensively studied in the past decades using different two-dimensional (2D) in vitro methods. These have allowed researchers to establish a model for cell migration that shows that an intermediate strength of adhesion is required for maximal cell speed (DiMilla et al., 1991; Palecek et al., 1997). This suggested that weak cell-adhesive interactions with the substratum would not provide enough traction and at strong adhesion, the cell would be too attached to the substratum to move efficiently. Interestingly, recent findings using three-dimensional (3D) in vitro and recently developed in vivo techniques have highlighted new features and intriguing differences in cell behaviour in comparison to what was previously described using 2D in vitro systems, namely in terms of cell morphology and signalling pathways controlling the process of cell migration (Baker and Chen, 2012; Beerling et al., 2011; Toetsch et al., 2009). As such, more and more researchers are turning to in vivo model systems (believed to be better mimicked by 3D in vitro systems) to clarify important aspects regarding cell migration.

*Drosophila melanogaster* has been a model organism of choice to look at cell migration in vivo. Border cell migration or epithelial cell migration, are some of best studied examples of collective cell migration (Montell et al., 2012; Weijer, 2009). To study single cell migration, immune circulatory cells, also known as hemocytes, which are equivalent to mammalian leukocytes in terms of functions and features, have emerged as a very useful model (Tirouvanziam et al., 2004; Wood and Jacinto, 2007).

Integrins are well-known transmembrane adhesion receptors involved in cell–extracellular matrix (ECM) interactions and play critical roles in cell signalling. They are one of the core components of adhesion sites where the ECM connects to the cellular cytoskeleton (Huttenlocher and Horwitz, 2011; Zaidel-Bar et al., 2007). The first step in adhesion establishment is the formation of nascent adhesions (NA), also referred to as focal complexes, which can then either disassemble or mature into focal adhesions (FA). The latter can undergo an additional maturation step which will give rise to fibrillar adhesions. In a study by Zaidel-Bar et al., the dynamics of the formation and maturation of these adhesion structures has been reported and some central differences in their composition have been shown (Zaidel-Bar et al., 2003). Although adhesion structures have been intensively studied in both 2D and more recently 3D in vitro systems (Fraley et al., 2010; Tolde et al., 2012), very little is known about their existence, formation and roles in an in vivo context. In *Drosophila*, the integrin family consists of five α_PS_ subunits (α_PS_1 to 5) and two β subunits, β_ν_ and β_PS_, the latter also known as *myospheroid* (Brown et al., 2000). The only previously reported links between hemocytes and integrins in *Drosophila* demonstrated that integrins act downstream of the GEF Dizzy and the Rap1 small GTPase to control both hemocyte shape changes and allow invasive migration during early embryonic development (Huelsmann et al., 2006; Siekhaus et al., 2010).

In this paper, we show that hemocytes that are located in dorsal patches, and are sessile during larval stages, depend on integrins and other known adhesion-related proteins to migrate after pupal formation. Whereas depletion of proteins described to be involved in NA formation leads to a decrease in hemocyte speed without affecting cell polarization, depletion of a recognized adhesion maturation-related protein increases cell speed. These findings suggest that the degree of maturation of the adhesions alters cell migration speed in a biphasic manner thus providing new molecular insights to further understand the biphasic model proposed by DiMilla et al., where an optimal adhesion strength maximises cell speed (DiMilla et al., 1991).

## Results

### Mutations in the *Drosophila* β_PS_ integrin subunit, *myospheroid*, lead to hemocyte migration defects

During late 3^rd^ instar larvae and early white pre-pupal stages of *Drosophila melanogaster* development, a dorsal population of hemocytes, hereupon referred to as dorsal patch-hemocytes, can be found attached to the dorsal epithelium aligned in periodic patches along the dorsal vessel (Lanot et al., 2001; Makhijani et al., 2011; Stofanko et al., 2008; Zettervall et al., 2004). During this period, hemocytes maintain a round morphology, a low migration speed and are unresponsive upon wounding of the epithelium (Babcock et al., 2008) in contrast to the hemocytes in embryonic (Stramer et al., 2005) or late pupal stages (Moreira et al., 2011). However, by 2 hours after pupa formation (APF), the dorsal sub-epidermal hemocytes have acquired a spread morphology with filopodium and lamellipodium-like structures and collectively initiate random single cell migration (supplementary material Movie 1).

**Fig. 1.**
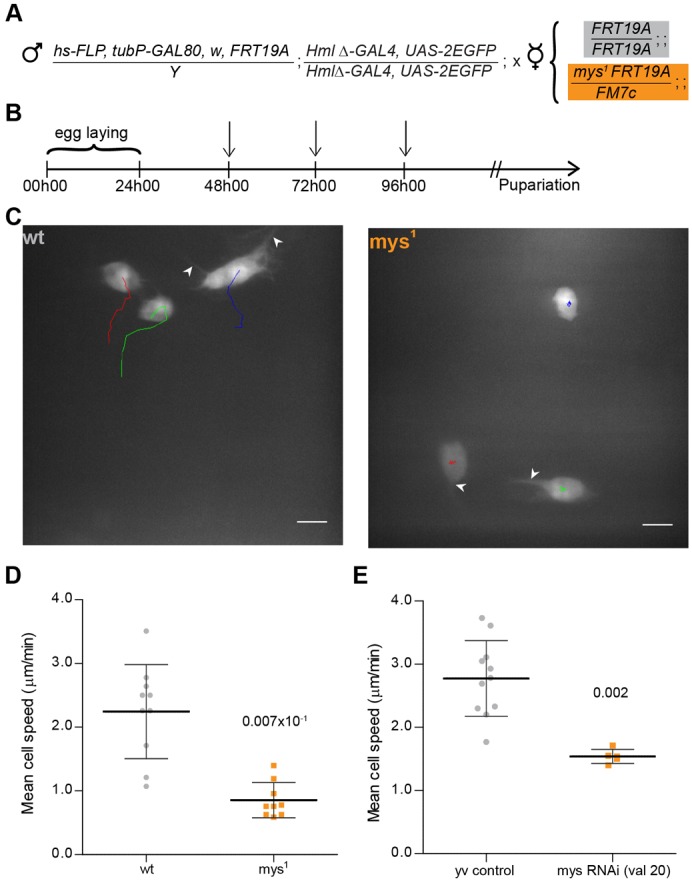
Myospheroid is required for proper hemocyte migration. (**A**) Outline of the MARCM protocol in hemocytes. Cross between DEMON males and *FRT19a* control or *mys^1^* mutant virgin female flies for MARCM analysis. (**B**) Crosses are placed at 25°C for 24 hours. The progeny is submitted to three 1 hour heat-shocks (indicated by arrows) at 37°C before selection of 3^rd^ instar females containing GFP-expressing hemocytes. (**C**) Movement of wild*-*type and *mys^1^* GFP-expressing hemocytes in 3 to 4 hour APF flies, tracked for 12 minutes (1 min time-lapse interval). Arrowheads indicate filopodium-like protrusions produced by both wild-type and *mys^1^* mutant hemocytes. Scale bars: 10 µm. (**D**) Graph showing individual mean cell speed for wild-type and *mys^1^* clones (2 to 4 hours APF). (**E**) Graph showing average mean cell speeds for *yv* control and *mys (valium 20)* RNAi-expressing flies (between 3 to 4 hours APF). Mann–Whitney tests for non-Gaussian populations were used. Black lines indicate the samples' means; Error bars  =  standard deviation; *P*-values shown above tested groups.

Based on the MARCM (**M**osaic **A**nalysis with a **R**epressible **C**ell **M**arker) system described by Wu and Luo (Wu and Luo, 2007), we established a protocol (DEMON – **De**leted in H**em**ocytes **On**ly) that allows to test the effects of homozygous lethal mutations in hemocytes only, in an otherwise phenotypic wild-type heterozygote background (Fig. 1A,B). Using this method we were able to generate GFP-positive wild-type and *myospheroid* mutant (*mys^1^*) hemocytes in the dorsal patches area (Fig. 1C), with a maximum of three GFP-positive cells in the selected region.

Between 2 and 4 hours APF, *mys^1^* hemocytes present striking differences in terms of cell movement when compared to controls (Fig. 1C,D): during this period, both wild-type and *mys^1^* hemocytes extend filopodium-like protrusions, typical of migrating cells. In terms of cell displacement however, it was evident that *mys^1^* hemocytes show an aberrant migratory behaviour. Calculation of individual cell speeds shows a significant difference between controls and *mys^1^* hemocytes with the former reaching an average speed of 2.24±0.74 µm.min^−1^ whereas the latter had a residual calculated mean cell speed of 0.86±0.28 µm.min^−1^. The movement in the *mys^1^* hemocytes is likely to be a consequence of the constant wiggling and cell shape changes that lead to permanent adjustments in the midpoint of the cell (supplementary material Movie 2). This result led us to hypothesize that, like in in vitro systems, other known adhesion components besides integrins, could play a role in dorsal patch-hemocyte migration upon pupal formation.

To test this hypothesis we decided to use the RNAi based methodology combined with the UAS/GAL4 system (Duffy, 2002) to specifically knockdown the genes of interest in hemocytes. Initially, we established that knockdown of *myospheroid* led to a significant decrease in cell speed (mean cell speed 1.54±0.11 µm.min^−1^) when compared to wild-type (mean cell speed 2.77±0.60 µm.min^−1^) in a similar manner as observed in the *mys^1^* hemocytes (Fig. 1E). This ensured that the RNAi based approach was suitable and sufficient for identifying anomalies in hemocyte migratory behaviour upon knockdown of genes of interest (supplementary material Movie 4). It is important to notice that in the case of RNAi knockdown some migration was still detected, compared to *mys^1^* mutant hemocytes, probably because this method does not result in a complete *mys* loss of function.

### Knockdown of integrins, integrin-activating and other adhesion-associated proteins affects hemocyte migration

**Fig. 2.**
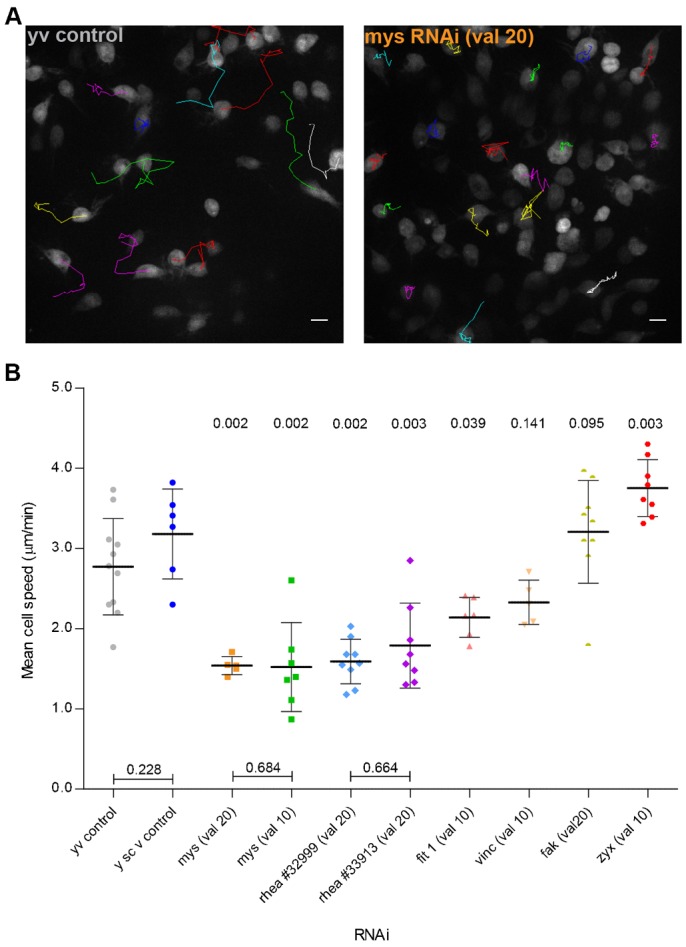
NA and FA proteins are essential for optimal hemocyte migration. (**A**) Example tracks of *yv* controls and *mys* RNAi-expressing flies. Scale bars: 10 µm. (**B**) Graph showing average mean cell speeds for different genetic backgrounds in 3 to 4 hour APF flies. val 10  =  TRIP valium 10; val 20  =  TRIP valium 20. Mann–Whitney tests for non-Gaussian populations were used. Both *rhea* RNAi lines were compared to the *y^1^sc^1^v^1^* control line. All other lines were compared to the *y^1^v^1^* control line. Black lines indicate the samples' means; Error bars  =  standard deviation; *P*-values shown above tested groups.

To understand the role of other integrin-related proteins known to be involved in the formation of NA (Choi et al., 2008; Zaidel-Bar et al., 2003) we decided to follow a candidate gene approach based protocol for specifically knocking down the genes of interest. Between 3 and 4 hours APF, whereas control hemocytes are already widely scattered cells, *myospheroid* deficient hemocytes remain closely clustered together forming a densely packed group of single cells (Fig. 2A; supplementary material Movies 3, 4). Two independent *mys* RNAi fly lines were used for comparison: a significant decrease in cell migration speed was observed with both (1.54±0.11 µm.min^−1^ and 1.52±0.56 µm.min^−1^ for the valium 20 and the valium 10 lines, respectively) (Fig. 2B). It is also interesting to notice that the patches form independently of the integrin knockdown.

Depletion of defined integrin-activating proteins such as Rhea (Talin homologue) or Fermitin 1 (Kindlin 1 homologue) (Anthis et al., 2009; Larjava et al., 2008) led to significant decreases in the mean cell speed (Fig. 2B; supplementary material Movies 5, 6). *rhea* knockdown reduces hemocyte cell speed to values comparable to the ones observed with the *mys* knockdown hemocytes (1.80±0.52 µm.min^−1^ and 1.59±0.21 µm.min^−1^ for the 33913 and the 32999 lines, respectively). *fermitin 1* knockdown, although to a lesser extent than *mys* and *rhea*, also causes hemocyte migratory defects, with the latter reaching average speeds of 2.14±0.25 µm.min^−1^.

Depletion of either Vinculin or Focal Adhesion Kinase (FAK), both described to be involved in early adhesion formation (Deramaudt et al., 2011; Zaidel-Bar et al., 2003), has no significant effect on hemocyte migration in vivo (Fig. 2B; supplementary material Movies 6, 7). These results correlate with previous findings which suggested that either molecule is non-essential for *Drosophila*'s viability (Alatortsev et al., 1997; Grabbe et al., 2004).

Following the stabilization of a NA, which in part involves the organized recruitment and assembly of the above discussed proteins, adhesion structures can develop into a FA, a process that relies on several factors, including tension which depends on myosin II activity (Parsons et al., 2010). This maturation process is accompanied by an increase in the number and diversity of proteins that assemble at adhesion sites, like zyxin. As such, we decided to investigate the effect of knocking down *zyxin* expression during hemocyte migration. Interestingly, *zyxin* knocked down hemocytes showed a significant increase in the mean cell speed (3.75±0.36 µm.min^−1^) when compared to controls (Fig. 2B; supplementary material Movie 7), a phenotype which has been previously described in in vitro cultured cells (Fraley et al., 2010; Hoffman et al., 2006).

### Polarization in response to wounds is not affected by the knockdown of NA related proteins

Integrins also appear as key molecules in the establishment and maintenance of a polarity axis in directionally migrating cells; therefore we decided to analyse their role in hemocyte polarization and migration upon epithelial wounding.

**Fig. 3.**
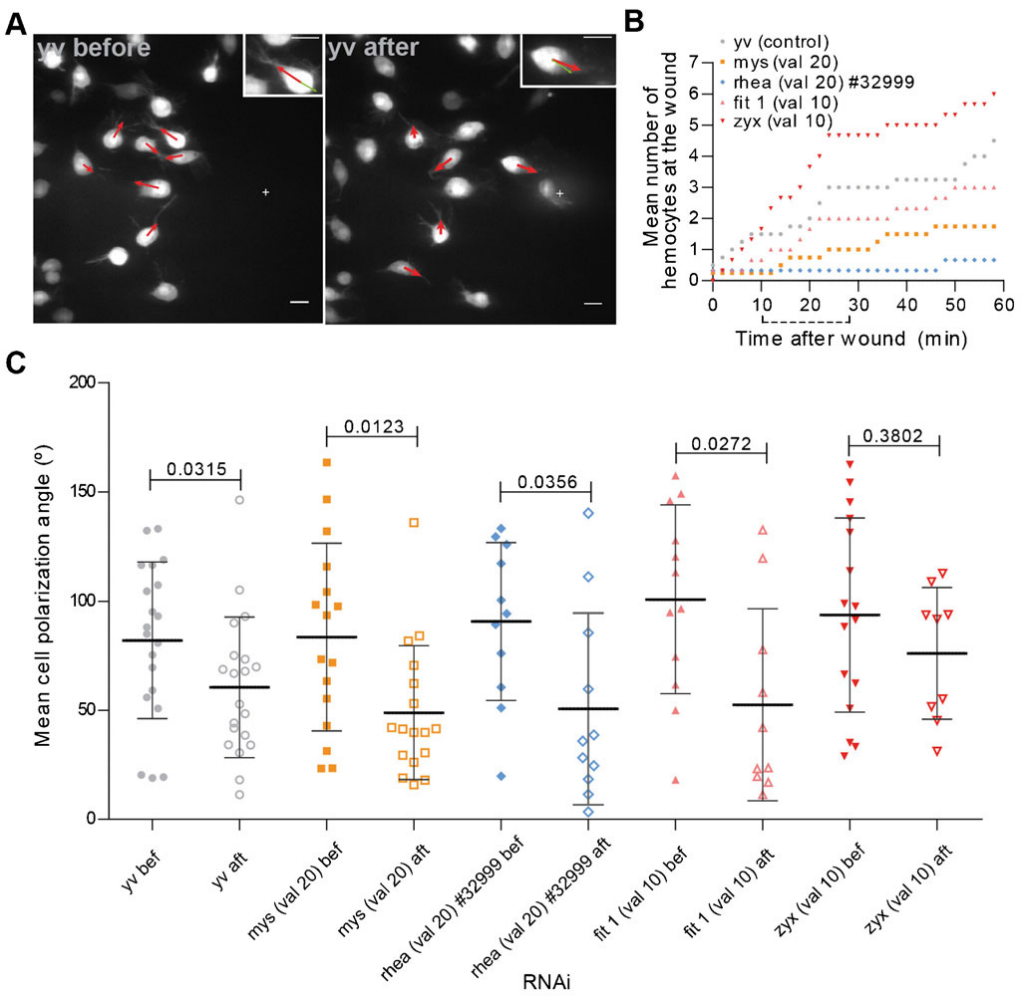
Polarization towards a chemotactic cue does not depend on NA proteins. (**A**) *yv* control flies immediately before and 28 minutes after wounding, respectively. Zoom of a selected cell presented at the top right hand corners, showing changes in the polarization arm. Red arrows point in the direction of the polarization arm of cells at the pictured time point. Green arrows point towards wound center (depicted by a white cross). Scale bars: 10 µm. (**B**) Graph showing the mean hemocyte numbers at the wound area along time for different control and RNAi flies. Dotted line under *x* axis depicts the chosen time interval for the polarization analysis. (**C**) Graph showing the mean polarization angle for individual cells before and after wounding in 3 to 4 hour APF flies. val 10  =  TRIP valium 10; val 20  =  TRIP valium 20. Black lines indicate the samples' means; Error bars  =  standard deviation; *P*-values shown above tested groups.

Three to four hours APF, the hemocytes in the dorsal patches area show a random migratory pattern with a filopodial protrusion extending in the direction of the migration. Upon wounding, part of the nearby hemocyte population responds by extending the filopodial protrusion towards the wound site (Fig. 3A; supplementary material Movie 8). Quantification of the number of hemocytes at the wound site over time in a control situation suggests that within the first 30 minutes after wounding, a first wave of rapidly responding hemocytes reaches the wound site (Fig. 3B). Over the next few minutes, some cells still respond to the chemotactic cue although the rate at which they reach the area of interest is diminished compared to the initial response phase. This behaviour is enhanced in *zyxin* knockdown hemocytes thus suggesting that blocking adhesion maturation does not affect hemocyte chemotaxis. For *myospheroid* and *rhea*-depleted hemocytes the response to a wound was less perceptible since only a few cells ever reach the area of interest during the time of analysis. However, since blocking the initial formation of NA leads to striking migratory defects, we decided to further characterize the hemocytes' response to a chemotactic cue: for that, we analysed the mean angle of the hemocytes' polarization arm relative to the wound before and after wounding (Fig. 3C). In the control population, we calculated a significant change in the mean angle of the polarization arm relative to the wound site. Interestingly, the knockdown of *myospheroid*, *rhea* or *fermitin 1* had no impact in the extension of the filopodial protrusion towards wounds: as for controls, in all situations, there was a shift to lower values of the mean angle of polarization, which is indicative of the arm pointing towards the wound site. This was observed independently of the capacity of hemocytes to migrate towards wounds (Fig. 3C; supplementary material Movies 9, 10, 11), thus suggesting that integrin-containing adhesions, although crucial for hemocyte migration, are not necessary for the establishment of polarity upon wounding.

In opposition to what happens with NA-related proteins, zyxin-depleted hemocytes showed no significant changes between before and after wounding in terms of the mean polarization angle (Fig. 3C; supplementary material Movie 12) which could suggest that *zyxin* knocked down hemocytes failed to reorient themselves towards the wound and/or were less sensitive to environmental cues. However, we have shown that the response of *zyxin*-depleted hemocytes occurs more rapidly than in a control situation thus probably making it a lot more difficult to detect shifts in the mean polarization angle as in all other analysed situations.

## Discussion

In this paper, we have looked at cell migration in vivo using *Drosophila* dorsal patches-hemocytes as our model system. These patches of immune cells are found attached to the dorsal epithelium aligned along the dorsal vessel of the late 3^rd^ instar and young white pre-pupas (Lanot et al., 2001; Stofanko et al., 2008). The origin of these tightly packed groups of cells is partially unknown, but it has been suggested that these serve as a major hematopoietic compartment in the larva that can be activated upon an immune challenge (Márkus et al., 2009). How these cells remain attached to the epithelium prior to the onset of metamorphosis is also not clear. Our results suggest that integrins (more specifically *myospheroid*, *Drosophila*'s main beta integrin subunit) and integrin-containing adhesions play no role in the original attachment of these hemocytes to the dorsal patches areas, thus indicating that alternative adhesions systems may also be involved in hemocyte attachment to these locations.

Interestingly, in spite of the numerous similarities between *Drosophila* hemocytes and mammalian leukocytes, we seem to have found what appears to be a striking difference in terms of their migratory behaviour: whereas vertebrate immune cells can move in complex in vivo, ex vivo and 3D in vitro environments in a integrin-independent fashion, using the force of the actin-network expansion (Lämmermann et al., 2008), *Drosophila* dorsal patch-hemocytes are dependent on integrin-containing adhesions to migrate. Hemocytes seem to therefore fit a more conventional model which describes integrin-containing adhesions as essential for cell migration (Huttenlocher and Horwitz, 2011). Besides *myospheroid*, we have also shown that *rhea* (*talin* homologue) and *fermitin 1* (*kindlin 1* homologue) depleted hemocytes suffer from similar migratory defects, both showing significant decreases in the mean cell speed, a phenotype that has been previously observed in HT-1080 *talin*-depleted cells in a 3D in vitro assay (Fraley et al., 2010). Also, both Talin and Kindlin 1 have been shown to bind directly to the β integrin cytoplasmic tail causing integrin conformational changes and promoting integrin activation (Harburger et al., 2009; Ye et al., 2011). Our data suggest that a similar integrin activation mechanism could occur in migrating hemocytes in vivo and that depletion of either molecule could impair the formation of NAs.

Vinculin is another core component of NAs (Zaidel-Bar et al., 2003). Vinculin binds directly to talin and the actin cytoskeleton (Humphries et al., 2007) thus acting both as a bridge and a force-transmitter (Grashoff et al., 2010). Disruption of vinculin in 2D cultured cells was shown to play a critical role in cell migration, leading to a significant increase of cell speed (Coll et al., 1995). However, more recently it was also shown that in a 3D environment, *vinculin* depletion decreases cell motility, a phenotype that was associated to a decrease in adhesion strength and lowering of the traction forces (Mierke et al., 2010). Our results in vivo show that vinculin depleted hemocytes suffer no significant changes in terms of cell speed when compared to controls, which is in line with previous findings by Alatortsev et al., who have shown that vinculin is non-essential for *Drosophila*'s survival and development (Alatortsev et al., 1997). As vinculin is one of the main force transmitters identified in integrin-containing adhesions, it has yet to be clarified whether other molecules could be playing this role in migrating hemocytes.

Focal Adhesion Kinase (FAK) is another molecule which plays a critical role in both NA formation and adhesion turnover, depending on the state of phosphorylation (Deramaudt et al., 2011). In mice, *FAK* depletion causes numerous defects that ultimately lead to embryonic death (Ilić et al., 2004). In *Drosophila*, *fak* expression was shown to be non-essential, and the absence of FAK has no effect on integrin-dependent mechanisms nor does it seem to influence border cell migration (Grabbe et al., 2004). Similarly, we observe that *fak* knockdown had no effect on hemocyte migration. *fak* over-expression however, has been shown to act negatively on integrin adhesion in *Drosophila*, and is potentially critical for adhesion remodelling. It would therefore be interesting to see if any migration defects would also occur upon over-expression of *fak* in hemocytes.

It is known that upon stabilization, NAs can overcome several maturation steps which imply the arrival of adhesion maturation-related proteins such as Zyxin to the adhesion sites. Zyxin is a stretch-sensitive mechanosensor (Yoshigi et al., 2005) important for the maintenance and repair of actin stress fibers (Smith et al., 2010) and it has been described as a molecular hallmark of the transition from an NA to an FA (Zaidel-Bar et al., 2003). We have shown that *zyxin* depletion enhances hemocyte migration speed, a phenotype which has already been shown by others and was correlated with an enhanced adhesion capacity that did not affect integrin expression levels (Fraley et al., 2010; Hoffman et al., 2006). Further analysis should elucidate whether adhesion strength and/or *myospheroid* expression are affected in *zyxin*-depleted hemocytes. Our results therefore suggest that *zyxin* knockdown may prevent the transition of NAs into FAs as in cell culture studies. Zyxin may be involved in the maintenance of hemocyte integrin activity to regulate migration. This is similar to the biphasic model (DiMilla et al., 1991), where an intermediate strength of cell–substratum interaction is essential for maximal cell migration. It is possible that the disruption of initial NA formation decreases cell adhesion and leads to a decrease in cell speed due to a lack of the necessary traction for migration. On the other hand, disrupting the maturation of FAs (stronger adhesive structures than NAs) decreases what should be a stronger cell–substratum interaction, and therefore causes an increase in cell migration. This suggests that cell adhesion maturation may be an important factor for controlling cell speed.

Immune cell migration, besides being a naturally occurring developmental process, can also be triggered by external environmental cues such as wounds or infections (Niethammer et al., 2009). Migration, a complex multi-step process, starts with the formation of an internal cellular asymmetry of molecules and structures that allows the establishment of a front–rear cell polarity that eventually translates itself in a directional movement towards the site of interest (Bornens, 2008; Li and Gundersen, 2008; Mellman and Nelson, 2008; Stramer et al., 2010). Amongst the many molecules involved in the establishment of this polarity axis are the integrins. Using a peviously described migration assay (Moreira et al., 2011), we were able to conclude that, in hemocytes, integrins and other NA-related proteins are not essential for the establishment of a polarization axis towards a wound. This is not the first report of immune cells responding to environmental cues in an integrin-independent fashion (Lämmermann et al., 2008), which implies that other receptors apart from integrins are functioning in detecting environmental changes and in establishing a front–rear polarity axis in dorsal patch-hemocytes.

The polarization results obtained upon *zyxin* knockdown suggested that *zyxin*-depleted hemocytes could be less sensitive to environmental cues, similar to what was described for zyxin-depleted fibroblasts in a haptotatic migration assay towards different integrin ligands (Hoffman et al., 2006). However, we have shown that zyxin-depleted hemocytes still reached the wound area at a higher rate than control cells within the first 30 minutes post wounding. Altogether, the data suggest that *zyxin* depletion solely affects cell migration speed without affecting a cell's response to a chemotactic cue.

Ultimately, our results clarify the importance of a tight regulation of adhesion formation and maturation for optimal cell migration in an in vivo context, which, similarly to the previously described biphasic model by DiMilla et al. based on cell adhesion (DiMilla et al., 1991), suggests that integrin-containing adhesions formation and maturation is the major force controlling hemocyte migration in vivo.

## Materials and Methods

### Fly stocks

w; hml^Δ^GAL4,UAS-GFP; (Sinenko and Mathey-Prevot, 2004) was used to visualize dorsal patches-hemocytes and to drive the expression of other UAS constructs in hemocytes specifically. The choice of a truncated version of the original hml driver by Goto et al. (Goto et al., 2001) relied on the hemocyte-specific pattern of expression that recovers all the wild-type hemocyte specific characteristics that had been lost in the original construct. To generate the DEMON stock the following stocks from the Bloomington Stock Center were used: *hsFLP*, *tubP-GAL80*, w*, *FRT19A*; *Pin*/*CyO*; (BL# 5133), *FRT19A*;; (BL# 1709), *mys^1^FRT19A*/*FM7c*;; (BL# 23862). For the migration and polarization analysis the following lines were used: *y^1^v^1^* (BL# 1509) and *y^1^sc^1^v^1^* (BL# 25710) were used as controls; *myospheroid* (valium 10) (BL# 27735), *myospheroid* (valium 20) (BL# 33642), *rhea* (valium 20) (BL# 33913), *rhea* (valium 20) (BL# 32999), *zyxin* (valium 10) (BL# 29591), *vinculin* (valium 10) (BL# 25965), fermitin 1 (valium 10) (BL# 25966), FAK (valium 20) (BL# 33617). Flies were raised on standard medium and maintained at 29°C until imaging, unless otherwise stated. Beginning of pupariation was identified according to what is described by Bainbridge and Bownes (Bainbridge and Bownes, 1981).

### MARCM clones generation

Crosses were maintained at 25°C between heat-shocks. Heat-shocks were carried out in a 37°C waterbath, followed by 1 hour at 18°C to extend G2 phase and improve MARCM efficiency.

### Live imaging

Live pupas were mounted as previously described (Moreira et al., 2011) except that the imaging was done directly through the transparent cuticle. For cell speed analysis, pupas were imaged in an Andor Revolution spinning disc confocal microscope (Andor Technology, UK) using a 40×/1.30 Plan Fluor PFS oil immersion objective (Nikon Instruments) in conjunction with a 488 nm OPSL CW laser and a 500–550 nm band-pass emission filter. For the polarization assay, pupas were imaged using a Zeiss LSM 5 Live line-scanning confocal microscope (Carl Zeiss, Jena) using a 40×/1.30 Plan-Neofluar oil immersion objective in conjunction with a 488 nm Sapphire laser and a LP 505 nm emission filter. Images and time lapses were analysed using Fiji software.

### Polarization assay

Pupas were imaged during 20 min (2 min interval) before wounding. Wounding was performed with a pulsed UV laser (355 nm) using a UGA-40 spot illumination scanning system (Rapp OptoElectronic, Germany) fitted to the line-scanning confocal microscope. Wounds were induced on six rectangle-forming spots on the pupa's dorsal epithelium close to the middle dorsal patch. After wounding, pupas were imaged during 1 hour.

To calculate the mean of the polarization angle, we chose three time points before and after wounding, per cell (time points 0, 10 and 18 min before wounding and time points 10, 18 and 28 min after wounding, respectively). The polarization angle corresponds to the angle formed between the wound center, the cell's center and its polarization arm. During migration, hemocytes can sometimes leave behind a trailing edge or present multiple long filopodium-like extensions that can resemble polarity arms. In such cases the cell displacement between flanking time points was used to determine which of the extensions was the leading protrusion. Only hemocytes in which polarity arms were clearly identified were used in the analysis.

### Image analysis and quantification

For cell speed quantification, the Manual Tracking and the Chemotaxis and Migration Tool 1.01 (Integrated BioDiagnostics) plugins were used. Maximum projections were used for the analysis. Hemocyte centers were marked manually and their coordinates used to calculate cell speed. A minimum of 15 cells per individual fly were used for these measurements. For polarization angle measurements, wound limits were manually marked and the geometrical center was selected as the wound center. The Angle tool from Fiji was used to measure the polarization angles according to what was previously described. A minimum of three individual flies for each genotype was used in the quantification. For counting the number of hemocytes at the wound area, only cells with protrusions touching the delimited wound area were considered.

## Supplementary Material

Supplementary Material
