# Supplementary material for: Drosophila integrin adhesion complexes are essential for hemocyte migration in vivo
Source: Biol Open. 2013 Jun 6;2(8):795–801. doi: 10.1242/bio.20134564 (PMC3744071; doi:10.1242/bio.20134564)
Supplement: Supplementary Material [file supp_2_8_795__index.html]

Drosophila integrin adhesion complexes are essential for hemocyte migration in vivo — Drosophila integrin adhesion complexes are essential for hemocyte migration in vivo — Supplementary Material 

# *Drosophila* integrin adhesion complexes are essential for hemocyte migration in vivo

## 

**Files in this Data Supplement:**

- Supplementary Material - Carolina G. A. Moreira et al. doi: 10.1242/bio.20134564
- Movie 1 - **Movie 1. y1v1 control hmlΔ-expressing dorsal patch-hemocytes migrating in a pupa, 3h32min APF.** Frames taken every 30 seconds using a 60× oil objective in a spinning disc confocal microscope. xy, xz and yz projections are shown. Scale bar: 10 µm in all dimensions.
- Movie 2 - **Movie 2. Wild-type and mys1 mutant MARCM/DEMON generated hmlΔ-expressing dorsal patch-hemocytes migrating in pupas, between 2 and 4 hours APF.** Frames were taken every 1 minute using a confocal microscope. Scale bars: 10 µm.
- Movie 3 - **Movie 3. y1v1 and y1sc1v1 control hmlΔ-expressing dorsal patch-hemocytes migrating in pupas, between 2h40min and 4 hours APF.** Frames were taken every 2 minutes using a spinning disc confocal microscope. Scale bars: 10 µm.
- Movie 4 - **Movie 4. y1v1control, myospheroid (val 20) and myospheroid (val 10) RNAi-depleted hmlΔ-expressing dorsal patch-hemocytes migrating in pupas, between 3h15min and 4 hours APF.** Frames were taken every 2 minutes using a spinning disc confocal microscope. Scale bars: 10 µm.
- Movie 5 - **Movie 5. y1sc1v1 control, rhea (val 20) # 32999 and rhea (val 20) # 33913 RNAi-depleted hmlΔ-expressing dorsal patch-hemocytes migrating in pupas, between 2h40min and 4 hours APF.** Frames were taken every 2 minutes using a spinning disc confocal microscope. Scale bars: 10 µm.
- Movie 6 - **Movie 6. y1v1control, fermitin 1 (val 10) and vinculin (val 10) RNAi-depleted hmlΔ-expressing dorsal patch-hemocytes migrating in pupas, between 3 and 4 hours APF.** Frames were taken every 2 minutes using a spinning disc confocal microscope. Scale bars: 10 µm.
- Movie 7 - **Movie 7. y1v1control, FAK (val 20) and zyxin (val 10) RNAi-depleted hmlΔ-expressing dorsal patch-hemocytes migrating in pupas, between 2h50min and 4 hours APF.** Frames were taken every 2 minutes using a spinning disc confocal microscope. Scale bars: 10 µm.
- Movie 8 - **Movie 8. y1v1 control hmlΔ-expressing dorsal patch-hemocytes migrating in a 3 hour APF pupa before (10 timepoints) and after (30 timepoints) wounding.** Frames were taken every 2 minutes using a confocal microscope. Scale bars: 10 µm.
- Movie 9 - **Movie 9. myospheroid (val 20) RNAi-depleted hmlΔ-expressing dorsal patch-hemocytes migrating in a 3 hour APF pupa before (10 timepoints) and after (30 timepoints) wounding.** Frames were taken every 2 minutes using a confocal microscope. Scale bars: 10 µm.
- Movie 10 - **Movie 10. rhea (val 20) # 32999 RNAi-depleted hmlΔ-expressing dorsal patch-hemocytes migrating in a 3 hour APF pupa before (10 timepoints) and after (30 timepoints) wounding.** Frames were taken every 2 minutes using a confocal microscope. Scale bars: 10 µm.
- Movie 11 - **Movie 11. fermitin 1 (val 10) RNAi-depleted hmlΔ-expressing dorsal patch-hemocytes migrating in a 3 hour APF pupa before (10 timepoints) and after (30 timepoints) wounding.** Frames were taken every 2 minutes using a confocal microscope. Scale bars: 10 µm.
- Movie 12 - **Movie 12. zyxin (val 10) RNAi-depleted hmlΔ-expressing dorsal patch-hemocytes migrating in a 3 hour APF pupa before (10 timepoints) and after (30 timepoints) wounding.** Frames were taken every 2 minutes using a confocal microscope. Scale bars: 10 µm.
