# Supplementary material for: Drosophila integrin adhesion complexes are essential for hemocyte migration in vivo
Source: Biol Open. 2013 Jun 6;2(8):795–801. doi: 10.1242/bio.20134564 (PMC3744071; doi:10.1242/bio.20134564)
Supplement: Supplementary Material [file supp_bio.20134564_bio.20134564-s1.pdf]

Biology Open

Supplementary Material

Carolina G. A. Moreira et al. doi: 10.1242/bio.20134564

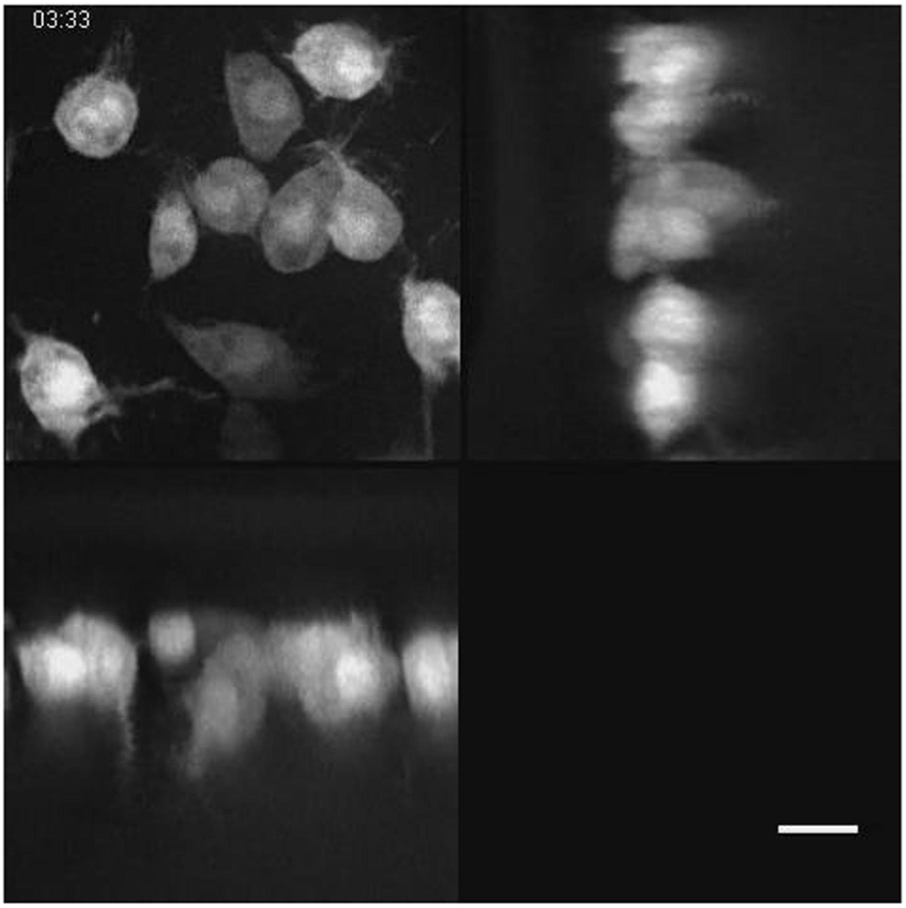

Movie 1. y^1v^1 control hml$^{\Delta}$-expressing dorsal patch-hemocytes migrating in a pupa, 3h32min APF. Frames taken every 30 seconds using a 60× oil objective in a spinning disc confocal microscope. xy, xz and yz projections are shown. Scale bar: 10 µm in all dimensions.

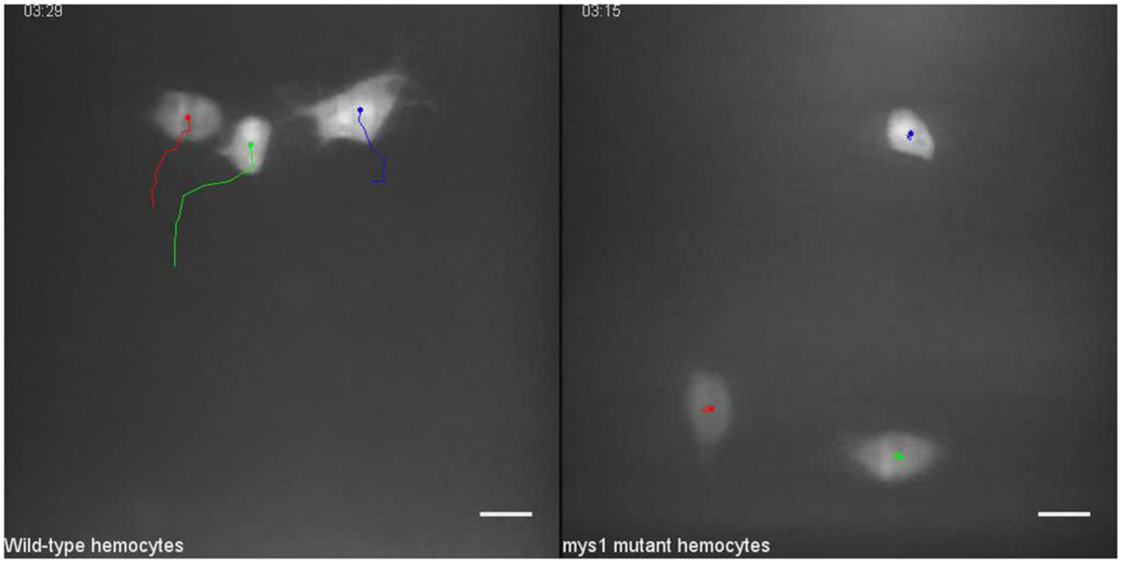

Movie 2. Wild-type and mys^1 mutant MARCM/DEMON generated hml$^{\Delta}$-expressing dorsal patch-hemocytes migrating in pupas, between 2 and 4 hours APF. Frames were taken every 1 minute using a confocal microscope. Scale bars: 10 µm.

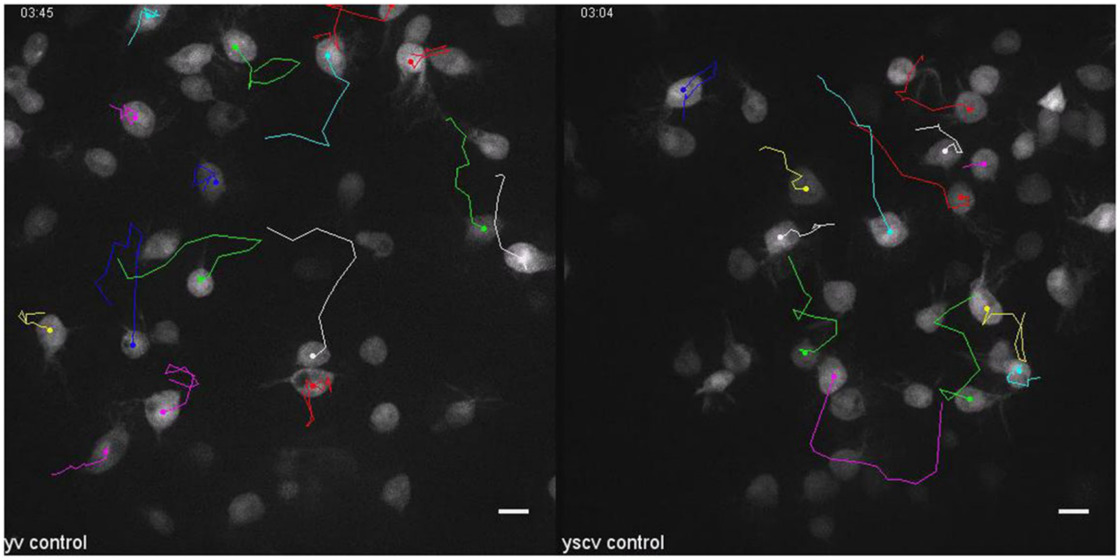

Movie 3. y^1v^1 and $y^1sc^1v^1$ control hml$^{\Delta}$-expressing dorsal patch-hemocytes migrating in pupas, between 2h40min and 4 hours APF. Frames were taken every 2 minutes using a spinning disc confocal microscope. Scale bars: 10 µm.

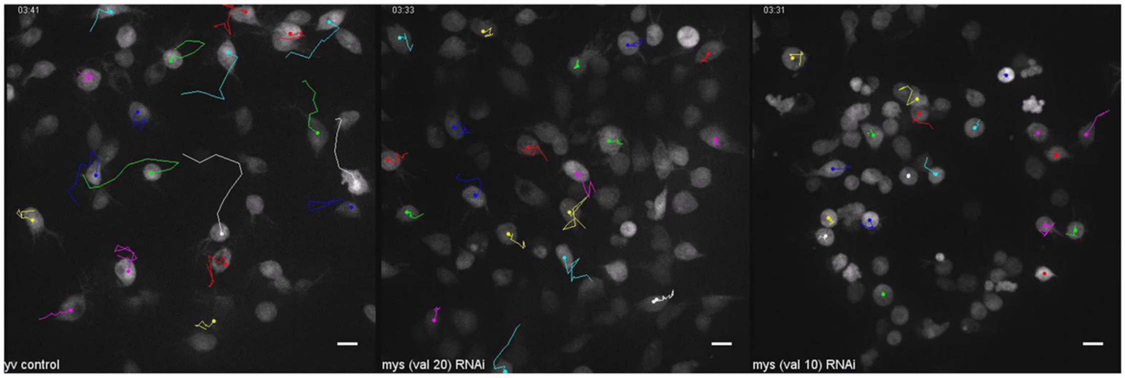

Movie 4. y^1v^1control, myospheroid (val 20) and myospheroid (val 10) RNAi-depleted hml$^{\Delta}$-expressing dorsal patch-hemocytes migrating in pupas, between 3h15min and 4 hours APF. Frames were taken every 2 minutes using a spinning disc confocal microscope. Scale bars: 10 µm.

Biology Open

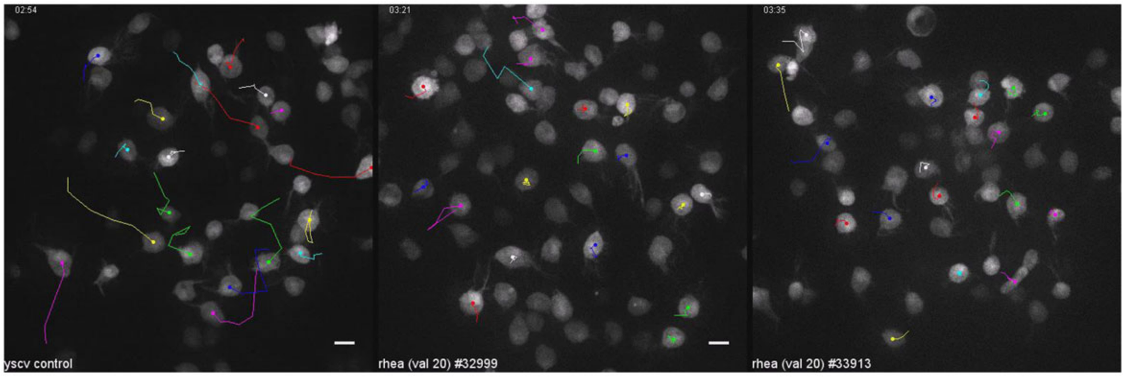

Movie 5. $y^{1}sc^{1}v1$ control, rhea (val 20) # 32999 and rhea (val 20) # 33913 RNAi-depleted hml$^{\Delta}$-expressing dorsal patch-hemocytes migrating in pupas, between 2h40min and 4 hours APF. Frames were taken every 2 minutes using a spinning disc confocal microscope. Scale bars: 10 µm.

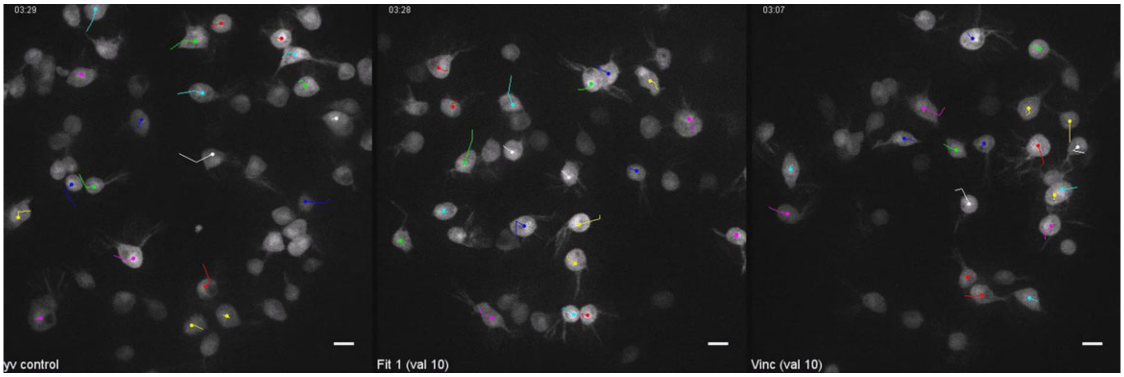

Movie 6. $y^{1}v^{1}$control, fermitin 1 (val 10) and vinculin (val 10) RNAi-depleted hml$^{\Delta}$-expressing dorsal patch-hemocytes migrating in pupas, between 3 and 4 hours APF. Frames were taken every 2 minutes using a spinning disc confocal microscope. Scale bars: 10 µm.

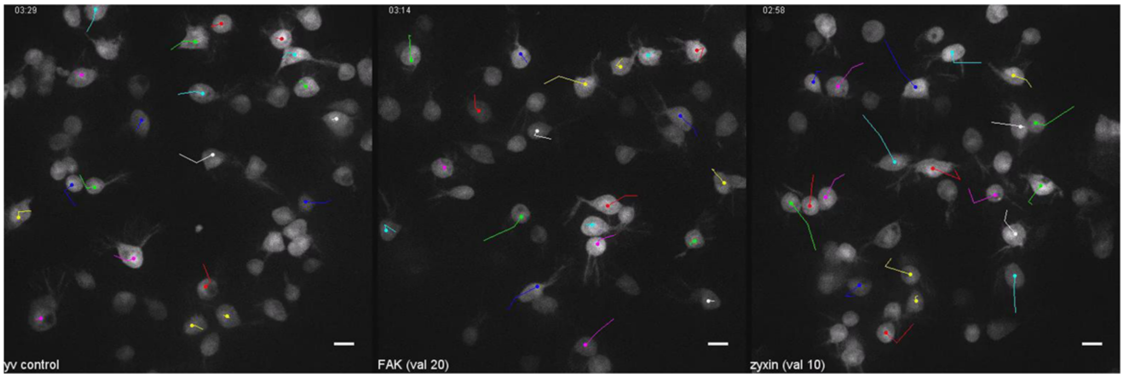

Movie 7. $y^{1}v^{1}$control, FAK (val 20) and zyxin (val 10) RNAi-depleted hml$^{\Delta}$-expressing dorsal patch-hemocytes migrating in pupas, between 2h50min and 4 hours APF. Frames were taken every 2 minutes using a spinning disc confocal microscope. Scale bars: 10 µm.

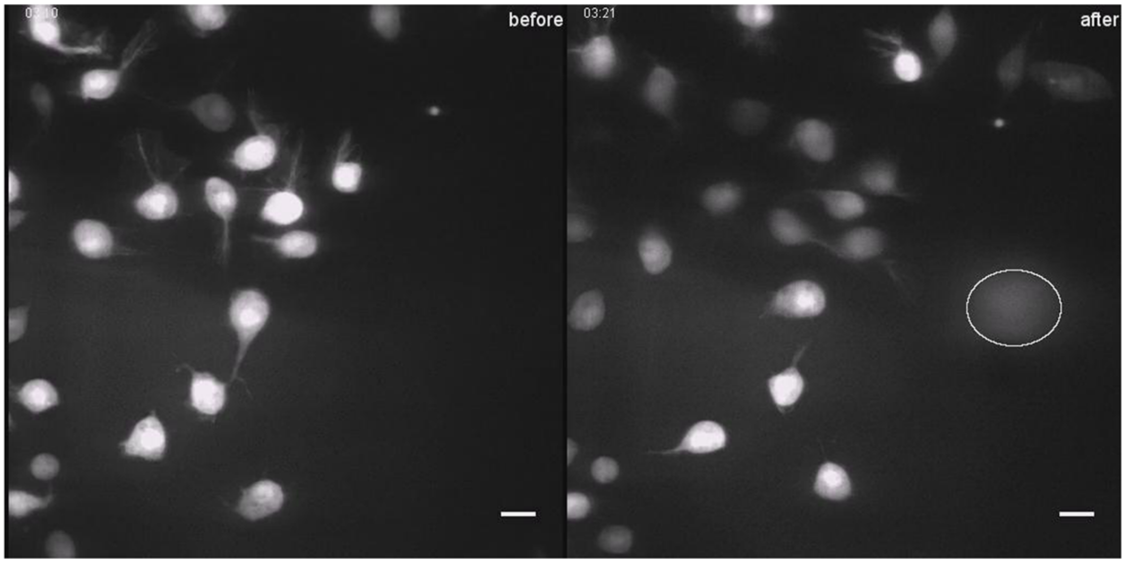

Movie 8. y[1]v[1] control hml$^{\Delta}$-expressing dorsal patch-hemocytes migrating in a 3 hour APF pupa before (10 timepoints) and after (30 timepoints) wounding. Frames were taken every 2 minutes using a confocal microscope. Scale bars: 10 µm.

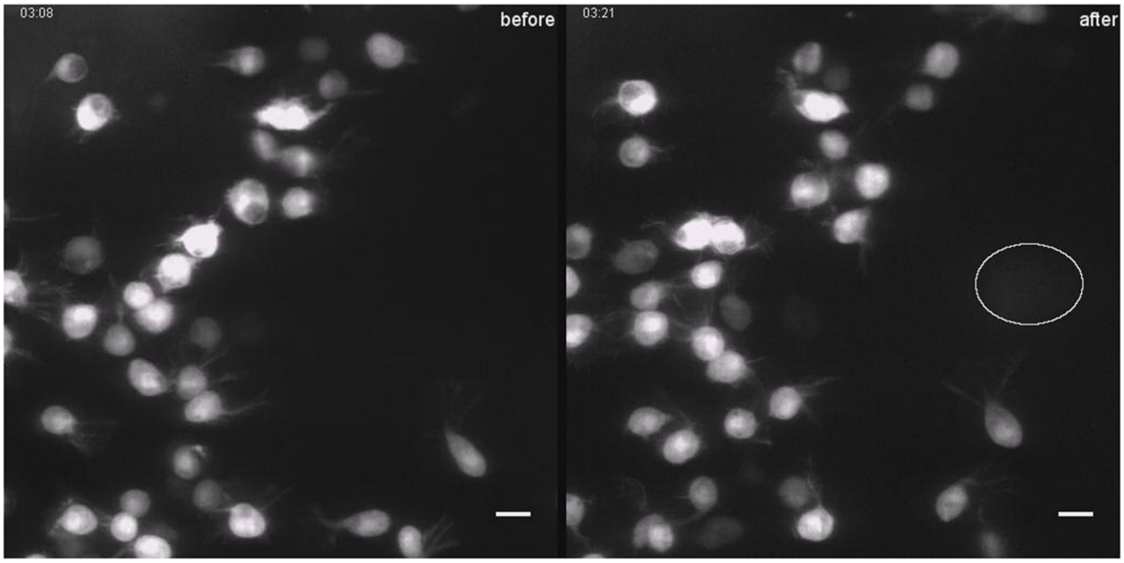

Movie 9. myospheroid (val 20) RNAi-depleted hml$^{\Delta}$-expressing dorsal patch-hemocytes migrating in a 3 hour APF pupa before (10 timepoints) and after (30 timepoints) wounding. Frames were taken every 2 minutes using a confocal microscope. Scale bars: 10 µm.

Biology Open

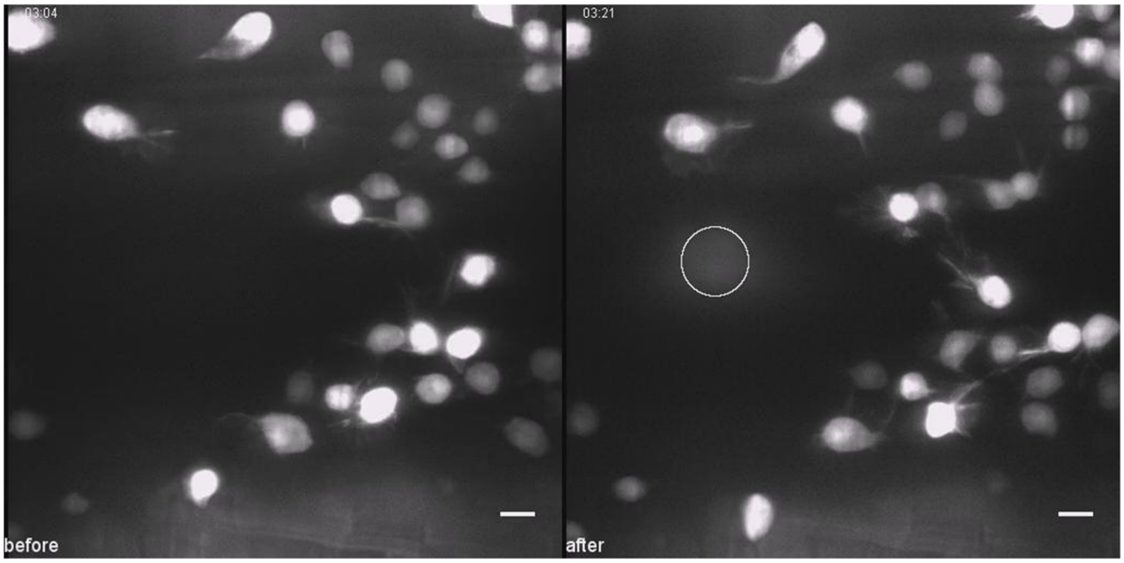

Movie 10. rhea (val 20) # 32999 RNAi-depleted hml$^{\Delta}$-expressing dorsal patch-hemocytes migrating in a 3 hour APF pupa before (10 timepoints) and after (30 timepoints) wounding. Frames were taken every 2 minutes using a confocal microscope. Scale bars: 10 µm.

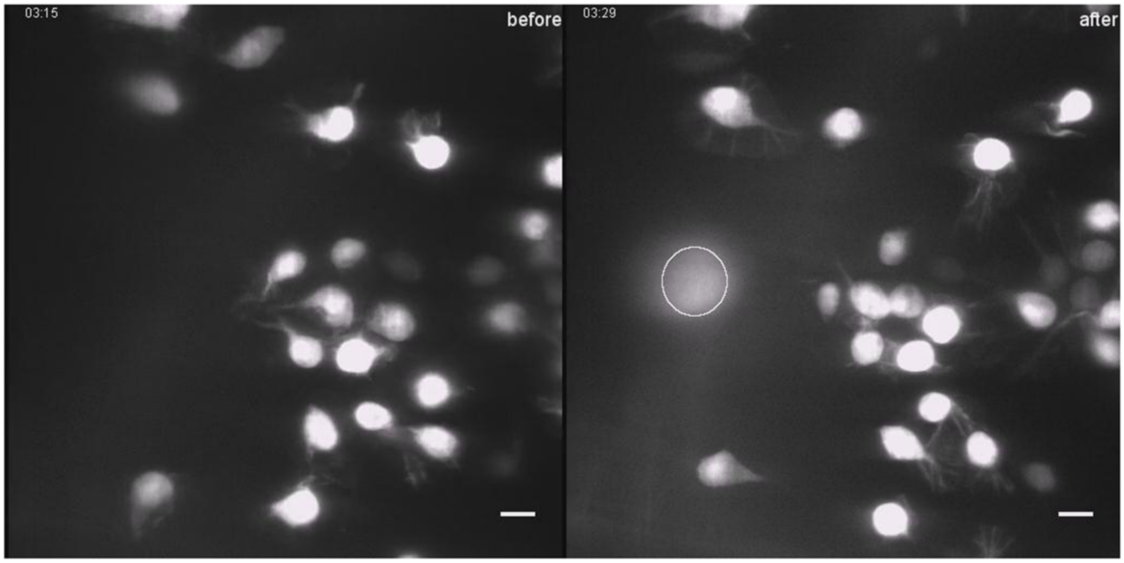

Movie 11. fermitin 1 (val 10) RNAi-depleted hml$^{\Delta}$-expressing dorsal patch-hemocytes migrating in a 3 hour APF pupa before (10 timepoints) and after (30 timepoints) wounding. Frames were taken every 2 minutes using a confocal microscope. Scale bars: 10 µm.

Biology Open

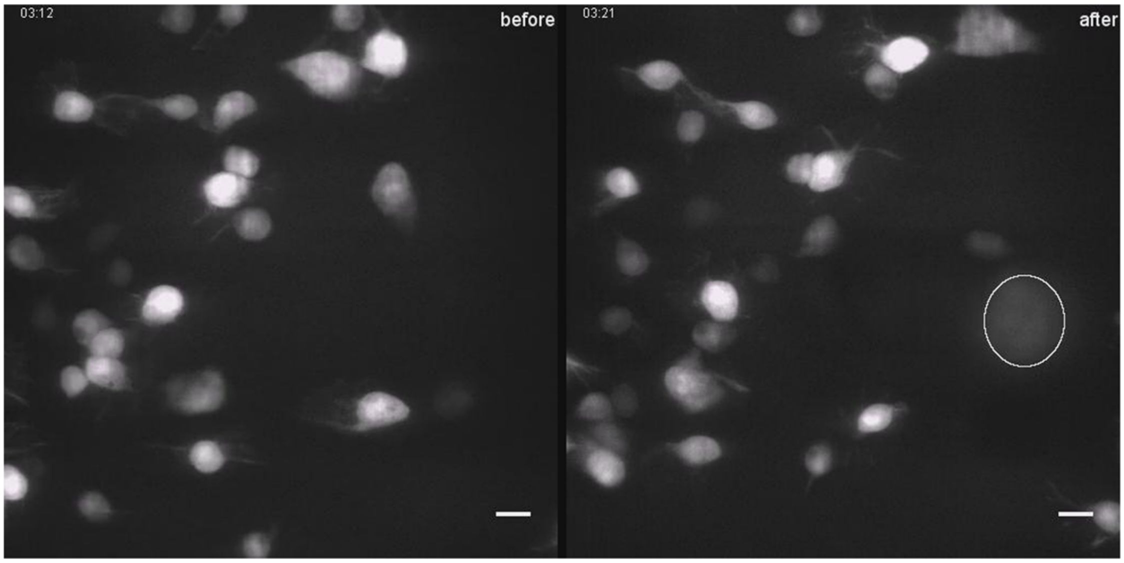

Movie 12. zyxin (val 10) RNAi-depleted hml$^{\Delta}$-expressing dorsal patch-hemocytes migrating in a 3 hour APF pupa before (10 timepoints) and after (30 timepoints) wounding. Frames were taken every 2 minutes using a confocal microscope. Scale bars: 10 µm.
